# Supplementary material for: Description of the embryonic development of Holothuria floridana (Pourtalès, 1851) to produce juveniles for aquaculture and restocking
Source: PLoS One. 2025 Jul 3;20(7):e0325564. doi: 10.1371/journal.pone.0325564 (PMC12225882; doi:10.1371/journal.pone.0325564)
Supplement: Attachment — Submitted filename: Response to Reviewers PONE-S-24-55150.pdf [file pone.0325564.s003.pdf]

Cinvestav

January 30th, 2025

Hector Escriva, PhD
Academic Editor
PLOS ONE

Thank you and the reviewers for the questions and suggestions about manuscript number PONE-S-24-70432, entitled "Description of the embryonic development of *Holothuria floridana* (Pourtalès, 1851) to produce juveniles for aquaculture and restocking."

We are including the responses to your doubts and queries. Accordingly, these revisions were made in the manuscript and tracked using the Word tracking system. A clean version of the revised manuscript is also provided.

RESPONSES TO THE EDITOR

1. **Q**: I recommend that the authors reproduce the experiments while addressing these critiques and include statistics and data on reproducibility so that the manuscript can be accepted.
2. **A**: Given that several sea cucumber hatchery manuals document the same methodology for other species (see the answer to Reviewer 2), the reproducibility of the method used here is achievable. For this reason, we did not consider repeating the experiment as a necessary condition for deeming this manuscript suitable for publishing.
3. **Q:** Please ensure that your manuscript meets PLOS ONE's style requirements, including those for file naming.
   A: It was revised that the manuscript meets PLOS NONE's style requirements

4. **Q**: In your Methods section, please provide additional information regarding the permits you obtained for the work. Please ensure you have included the full name of the authority that approved the field site access and, if no permits were required, a brief statement explaining why.
   A: Thank you for this recommendation. We have made the necessary edits to include the government department that assisted us. Regarding permits, this kind of study does not require special licenses in Mexico, considering that we did not conduct experiments that compromised the life, health, or well-being of the broodstock we worked with. The broodstock was collected by personnel from the Mexican Institute for Research in Sustainable Fisheries and Aquaculture, the country's authority regulating this activity. The national authority provided the sea cucumbers for scientific purposes in aquaculture activities, with the consideration of returning it to the collection site at the end of the spawning season.

5. **Q**: In the online submission form, you indicated that The data supporting this study's findings are available from the corresponding author upon reasonable request

A: Thank you for this recommendation. We are sharing the data as supplementary information.

RESPONSES TO REVIEWERS

Reviewer 1

1. **Q**: The abstract currently describes the methods used in the study and known results from previous work rather than highlighting the study's key findings and conclusions. The authors should rewrite the abstract to help the reader grasp at first sight the context and interest of the study.
   A: Thank you for this recommendation; we have rewritten the Abstract accordingly

2. **Q**: The introduction lacks mention of knowledge from already published work *on H. floridana*, including the fact that histological studies on male and female tubules, as well as some images of fertilized oocytes, blastulae, and pentactulae, have already been published and provide some conclusions. This is an important piece of information that should be added to the introduction in addition to information on what the current study is adding as new knowledge.
   A: The introduction includes brief information related to the *H. floridana* female's gonads histology and maturation stages, but describing the histology and maturation cycle of the species was not an objective of this study. Considering that recent studies describe these in detail, we do not include a histology literature review in the introduction of this paper.

3. **Q**: A table, histogram, or any kind of graphical representation would help visualize and understand the oocyte density distribution described in the result section "Histology of female gonads"
   A: Thank you for this recommendation. We did not include this update in the manuscript for the following reasons: a) Documenting female gonad maturation was not the main objective of this study; b) We documented female gonad ripeness to ensure that the oocytes were mature and viable for the study, and histology confirmed that the collected sea cucumbers were ripe enough to reproduce, and, c) Ramos Miranda et al. (2017 [16]) made a comprehensive summary of female gonad maturation, and we did not want to repeat what has already been published for *H. floridana*. However, we have included a table to clarify these results, indicating in Fig 1 the different phases observed.

4. **Q**: On page 11, the caption for Figure 3 is likely incorrect. Although B looks like it illustrates indeed the "first cell division until the formation of the two blastomeres", C likely shows an embryo after the second cell division, D after the third, and so on.
   A: Captions have been edited accordingly.

5. **Q**: On page 12, lines 243-244, the authors mention, for the first time, the presence of the intestine, while in Figure 5 (and its related caption), they indicate the presence of the intestine starting at 5 DAF. Please correct the statement or figure caption appropriately.

A: The information in the text was corrected to match what is indicated in the figure.

6. **Q**: On page 13, lines 268-271, the references to the images of Figure 7 should be corrected. As two examples, Fig 7 should read Fig 7A and Fig 7D should read Fig 7C. **A**: The missing information related to the description of the different images was included in the text.

7. **Q**: On page 15, in the caption for Figure 5, the authors should define what is the annular canal and pinpoint it in Figure 5C.
   A: "Annular canal" was changed in the text to the correct denomination (ring canal) and included its acronym (rc) in the caption

8. **Q**: On page 16, the authors mention on several occasions the presence of the intestine from juveniles 9 DAF to 20 DAF. It would be nice if they could provide an image showing the intestine at some of these stages in Figure 10.
   A: Numbers in the figures have changed. In Figure 9, images showing the intestine on days 9, 10, 15, 20, 30, and 45 after hatching are included, and the same is possible to observe in Figure 4. These were the only days when histology was made.

9. **Q**: In the caption for Figure 5, the stomach is abbreviated "e" while in the captions for Figures 9 and 10, it is abbreviated "st". For consistency, please abbreviate similarly stomach through all figures (and the same applies to any other abbreviated terms).
   A: The abbreviation in Figure 4 (former 5) was changed for consistency with the now Figures 8 and 9

10. **Q**: In the caption for Figure 5, the text "Juvenile of 20 DAF: sigmoid intestine and anus." should read "Juvenile of 20 DAF: sigmoid intestine and anus (a)."
    A: Thank you for the suggestion; it was corrected.

11. **Q**: In the caption for Figure 8, "arquenteron" should read "archenteron" and "24-h" should read "24 HAF".
    A: Thank you for the suggestion; it was corrected.

12. **Q**: In the caption for Figure 9, the text "ring canal of the water vascular system." should read "ring canal of the water vascular system (rc).
    A: Thank you for the suggestion; it was corrected.

Reviewer 2

1. **Q**: The authors show *H. floridiana* development starting from the ovaries content and fertilized oocytes to the advanced juvenile stages, while the description of the efficacy of the spawning method is poorly presented. The authors do not mention how many times they have repeated the spawning induction and what was the overall success rate of spawning. This is an important point if the aim of the work is to develop protocols for aquaculture.
   A: The induction of spawning of *H. floridana* using thermal shock is a method widely applied worldwide with other sea cucumber species (see Agudo, 2006:

https://www.aciar.gov.au/sites/default/files/legacy/node/758/Sandfish%20hatchery%20techniques%20%28english%29.pdf); Al Rashidi et al., 2012: https://scholar.google.com/citations?view_op=view_citation&hl=en&user=umXXeucAAAAJ&citation_for_view=umXXeucAAAAJ:W7OEmFMy1HYC; Altamirano & Rodriguez, 2022 https://www.researchgate.net/publication/363271266_Hatchery_production_of_sea_cucumbers_sandfish_Holothuria_scabra); Ito, 2018: https://www.ctsa.org/files/publications/SeaCucumberHatcheryManual.pdf).

This method has been regularly used in our lab and a hatchery in Panama to reproduce this species, at least during the last five years. It is applied to the selected broodstock on days coinciding with the new and full moon. Around 10% of treated broodstock respond to the stimulus in each induction, but the same female can spawn several times during the reproductive season (see Felaco et al., 2024: https://doi.org/10.1016/B978-0-323-95377-1.00008-4). Even though the induction was not an objective of this work, we have edited the section and added new information to answer this comment.

2. **Q**: In addition, the main developmental stages for this species have already been described in previous works, and the detailed description, including all the histological observations, is not indispensable to developing protocols for reproduction and rearing for aquaculture. On the other side, this detailed description could be useful for developmental biologists. The fast development and the absence of a planktonic larval stage before the formation of the juvenile make this system potentially useful for studying organogenesis and the evolution of pentameric structures in echinoderms. However, the authors made the description of development on embryos generated from oocytes deriving from one female, making the description and timing of the different stages presented in the figures and Table 1 not robust.
A: We do not know a description of the embryonic development previously published with the details that we are offering. Documenting female gonad maturation was not an objective of this study. Still, it was reviewed to confirm that the collected sea cucumbers were ripe enough to reproduce and ensure that the oocytes were mature and suitable for the study.
Besides that, our histological description gives those interested in the culture of this species a better understanding of the development of the different organs of interest, especially the digestive tract and respiratory tree. This information is essential to developing farming systems and protocols for handling and feeding pentactula and juveniles in the hatchery, giving the possibility of moving early stages towards intermediate growing systems directly to hapas in the wild as early stages as possible.
On the other hand, the embryonic development description will be the same using oocytes from one or several females. We do not expect differences if we use oocytes from one or various females or spawn or repeat this study several times using different females, which is time-consuming and expensive. The description was made using slides from several oocytes, all showing the same.

3. **Q**: The overall terminology used to describe the different phases of development is in many cases not appropriate and some sections are not clearly presented. Examples are the use of the word "organisms" when referring to the sea cucumber adults, embryos and larvae.
   A: Thank you for the observation, which was attended to name the different stages accordingly.
4. **Q**: Also, some headings in the methods sections are not appropriate, for examples "Embryonic development" and "Organogenesis" are more appropriate for a Results section and should be "Embryo cultures, fixation and imaging" and "Hystological analysis" respectively.
   A: Thank you for the suggestion; it was considered in the text when applicable.
5. **Q**: Figures are not clear, adding more arrows could help the reader to identify the objects described in the text, for example: in Fig. 1 the authors refer to mature oocytes and degraded oocytes without pointing at them in the figures. This is true for most figures. Also, time of development in hpf and stages of development should be included in all the figures panels to help the reader.
   A: Thanks for your observation. The suggestions have been accounted for.

Several English mistakes and wrong wording are present all over the manuscript, examples:

6. **Q**: lines 48-49: "Overfishing sea cucumbers has led to declining or disappearing wild populations, considering aquaculture as an alternative to meet its commercial demand" should be "Overfishing sea cucumbers has led to declining or disappearing wild populations, making aquaculture an alternative to meet its commercial demand"
   A: Thanks for your observation. Your suggestion was considered in the writing
7. **Q**: line 65: "They have an abbreviated lecithotrophic larval development" should be "They have a fast lecithotrophic larval development"
   A: Thank you for the observation; the wording was modified accordingly.
8. **Q**: line 111: "For this, samples of organisms were placed in a vial containing 2.5 mL of seawater and 2-3 drops of clove oil in seawater" should be "For each time point embryos, larvae and juveniles were placed in a vial containing 2.5 mL of seawater and 2-3 drops of clove oil in seawater."
   A: The suggestion was attended to.
9. **Q**: line 153: "weighed" should be "weighted"
   A: Weighed is the correct expression for obtaining the weight of something
10. **Q**: line 154 "Conformed" should be "formed"
    A: The observation was attended to.
11. **Q**: line 163: "vitellogenic oocytes were observed…" should be replaced with "oocytes"
    A: The suggestion was agreed to.
12. **Q**: line 168-169 is not clear and should be refrased

A: The observation was attended to.

13. **Q**: the abbreviations used for the statistics should be explained
A: The suggestion was attended to.

14. **Q**: lines 206-210: the authors state that the adhesive gelatinous capsule gets thicker after fertilization, however this is not shown in figure 2 and it is not presented in terms of results of the measurements (they only measure it after fertilization). They also refer to panel C of figure 2 to introduce the oocyte protuberance (that, by the way, they don't define) while this is visible in panel B. In general, I don't see figure 2 informative, it could be fused with figure 3.
A: There was an error in writing “thicker” instead of “thinner.” As shown in Figure 2, the gelatinous capsule decays during embryonic development, and this process is described in the text.
Regarding Figure 2, omissions were attended, and that figure was merged with Figure 3 as suggested.

15. **Q**: line 212: "sowing" should ne "showing"
A: The observation was attended to.

16. **Q**: line 220: "A: formation of the polar body" is wrong, in the figure they show the extrusion of the polar body after fertilization; polar bodies form during oogenesis.
A: The observation was attended to.

17. **Q**: HAF (hours after fertilization) and DAF (days after fertilization) should be replaced with the broadly utilized hpf and dpf (hors post fertilization and days post fertilization)
A: The suggestion was attended to.

18. **Q**: line 241: "a main podia" should be "a main podium"
A: The suggestion was attended to.

19. **Q**: line 284: the title "Organogenesis" should be "Embryonic development and organogenesis"
A: The suggestion was attended to.

20. **Q**: line 297 “arquenteron” should be “archenteron”
A: The suggestion was attended to.

21. **Q**: the Discussion section needs major English corrections
A: Thanks for your comments on the English wording, which has been considered, mainly the typos. However, I would like to remark that the original and updated manuscript was extensively reviewed by a native English speaker, Dr. Arlenie Rogers, a researcher at the University of Belize and co-author of the article.

Dr. Miguel A. Olvera-Novoa
Corresponding author
