# Supplementary material for: Description of the embryonic development of Holothuria floridana (Pourtalès, 1851) to produce juveniles for aquaculture and restocking
Source: PLoS One. 2025 Jul 3;20(7):e0325564. doi: 10.1371/journal.pone.0325564 (PMC12225882; doi:10.1371/journal.pone.0325564)
Supplement: Attachment — Submitted filename: Rebuttal_letter PONE-D-24-55150R1.pdf [file pone.0325564.s004.pdf]

**Centro de Investigación y de Estudios Avanzados del IPN**
**Unidad Mérida**
**Departamento de Recursos del Mar**

April 11, 2025

**Hector Escriva, PhD**
**Academic Editor**
**PLOS ONE**

In this document, we detail the attention given to the suggestions and questions made to our first revision of the article "Description of the embryonic development of *Holothuria floridana* (Pourtalès, 1851) to produce juveniles for aquaculture and restocking" (Manuscript ID: PONE-D-24-55150R1) for consideration for publication in *PLOS ONE*.

## RESPONSES TO THE EDITOR

1. We reviewed the reference list to ensure it was complete and accurate.
2. We added a new citation, including an article describing echinoderm oogenesis, to help address reviewers' concerns and included it in the reference list.
3. The numbering in the manuscript and the reference list was updated to reflect the inclusion of the new citation.
4. We attended to the reviewers' observations and recommendations, including our responses below.

## RESPONSES TO REVIEWERS

| # | Question | Reply |
|---|---|---|
| 1 | In the results section entitled "Histology of female gonads" the authors should clarify the difference between "vitellogenic oocytes" and "mature oocytes" for non-specialists. If there is no difference between the two terms, the authors should always use the same term to avoid confusion. Also, in this section, the authors should indicate the type of oocytes they counted to determine the density and frequency of oocytes in the ovaries (i.e. vitellogenic oocytes only, mature oocytes only, or all oocytes including vitellogenic, mature, primary, and degrading oocytes). | This research publication is intended for a specialized scientific audience familiar with the subject matter. As such, we do not consider it necessary to include definitions of technical terms within the text. However, for readers seeking clarification, we have provided a reference (Smily, 1990) where detailed definitions can be found.<br>This is the reference:<br><br>Smiley, S. (1990). A review of echinoderm oogenesis. *Journal of electron microscopy technique, 16*(2), 93-114. |
| 2 | The beginning of the results section entitled "Embryonic development" includes some inconsistencies. From the text and the images provided, it sounds as if all images in Figure 1 correspond to fertilized oocytes and subsequent embryos. If so, Figure 1A | Thank you for this comment. We have revised and reworded the Figure 1 caption and moved the figure caption below the entire paragraph. |

| | | |
|---|---|---|
| | illustrates indeed the fertilization envelope, but which surrounds the fertilized 1-cell stage embryo and not simply some yolk. Likewise, the protuberance seen in Figure 1B is at the level of the fertilization envelope and not the oocyte. The authors should revise their figure caption and results section based on this comment. Also, the figure caption has several typos that should be corrected. | |
| 3 | Still, in the results section entitled "Embryonic development", the authors should clarify, for non-specialists, the morphological difference between a morula and a blastula. This will help produce practical information for developing hatchery infrastructures. Also, the formation of a blastocoel is not conventionally accepted as a sign of invagination. The formation of a blastocoel is what characterizes the blastula stage. Invagination means the movement of tissues within the blastocoel, which is the mark of gastrulation and takes place as described here between 12 hpf and 24 hpf. The authors should thus correct their descriptions of these stages in the text and the figure caption.<br><br>In Figure 3B, it is possible also that the embryo exhibits already a blastopore. If this is correct, this structure should be highlighted in the figure and mentioned in the text and the figure caption.<br><br>Last, in the caption of Figure 3, the authors outline the presence of "primary oral tentacles", "oral tentacle primordia" and "defined oral tentacles" when, in the text, they first mention the presence of "primordia of the oral tentacles" and then "primary oral tentacles" but never refer to "defined oral tentacles". The authors should provide consistent naming of the tentacles according to their level of development and homogenize the text and figure caption accordingly. | With regards to the comment on the definition of morula and blastula: to maintain the focus and conciseness of the manuscript, we have opted not to include basic definitions. Instead, an in-text citation has been provided, allowing readers to consult the referenced source for terminology clarification if needed.<br><br>With regards to the comment on the formation of the blastocoel and gastrulation: we have edited the text to include this corrected information.<br><br>Regarding Figure 3B, we reviewed our files from all other embryonic development observations made in Mexico, Belize, and Panama and concluded that the blastopore does not appear at this stage.<br><br>Regarding the captions in Figure 3, we have corrected the text and the captions accordingly. |
| 4 | In Figure 4, the authors indicate in panel F the presence of an anus. This notion should be indicated in the text as well. Likewise, in the | Thank you for this comment. We have edited the caption and text accordingly. |

| | | |
|---|---|---|
| | text, the authors highlight the increasing number of oral tentacles and secondary podia as a characteristic of the different juvenile stages they observed. They should pinpoint these tentacles and podia in Figure 4 panels G to I. | Figure 4, panels G to I, were edited accordingly. |
| 5 | On pages 18 and 19, in the paragraph discussing the histological observations, the authors write that, in their study, they observed a gonad in "the development stage" hence stage II and a gonad "classified at stage V" hence post-spawning. Yet, in the results section, the authors indicate that one of the gonads they studied was "in Stage III: Mature". The authors should revise this inconsistency. | Thank you for this comment. We have edited the text and the figure accordingly on page 10. |
| 6 | Still in the discussion, in lines 473-475, the authors indicate: "Unlike planktotrophic species, holothuroids with vitellaria lecithotrophic larvae do not proceed through the auriculara and doliolaria phases". Yet, in the sentence that just follows, they wrote: "they go directly from the gastrula to doliolaria-type larvae [19, 43]". This sounds incoherent. Maybe, there is a typo issue. | Thank you for this comment. We have edited the text to make it coherent. |
| 7 | - In line 33, "aqueous vascular system" should read "water vascular system".<br><br>- In line 204, "in short and long ovary tubules and the sections anterior(A)," should read " in short and long ovary tubules and in anterior (A),".<br><br>- In line 229, please amend "with spawning" to "with egg spawning".<br><br>- In line 255, a verb is likely missing in the sentence "as the primordia of the oral tentacles".<br><br>- In line 366, the sentence "the histology of fertilized oocytes allowed the observation of" should read "the histology of samples post- | Thank you for these comments. We have:<br><br>- Replaced the word "aqueous" with "water"<br>- Edited the text to read "in short and long ovary tubules and in anterior (A),".<br>- We did not amend the text to include "with egg spawning" as this paragraph speaks about spermiation in the same sentence and should, therefore, not include "eggs".<br>- We have edited the text to fix the verb in the sentence "as the primordia of the oral tentacles"<br>- We edited the text to include "the histology of samples post-fertilization allowed the observation of". |

| | | |
|---|---|---|
| | fertilization allowed the observation of".<br><br>- In line 325, the caption title for Figure 7 should read "Histological sections of H. floridana embryos".<br><br>- In line 356, "At 20 days post-fertilization (dpf)" should read "At 20 dpf"<br><br>- In lines 481-482, the mention of "do not feed" is redundant with "lecithotrophic". | - The caption of Figure 7 has been edited to include "Histological sections of *H. floridana* embryos".<br>- The text has been edited to include "At 20 dpf"<br>- The words "do not feed" have been removed from the sentence. |

Sincerely,

Dr. Miguel Angel Olvera Novoa
Centro de Investigación y de Estudios Avanzados
[Center for Research and Advanced Studies]
miguel.olvera@cinvestav.mx

On behalf of all co-authors
